# PCSK9 inhibition: from effectiveness to cost-effectiveness

**DOI:** 10.3389/fcvm.2024.1339487

**Published:** 2024-06-25

**Authors:** Iveta Mercep, Dominik Strikic, Pero Hrabac, Ivan Pecin, Željko Reiner

**Affiliations:** ^1^ Department of Internal Medicine, School of Medicine, University of Zagreb, Zagreb, Croatia; ^2^ Division of Clinical Pharmacology, Department of Internal Medicine, University Hospital Centre Zagreb, Zagreb, Croatia; ^3^ School of Medicine, University of Zagreb, Zagreb, Croatia; ^4^ Department of Internal Medicine, University Hospital Centre Zagreb, Zagreb, Croatia; ^5^ Department of Cardiology and Congenital Diseases of Adults, Polish Mother’s Memorial Hospital Research Institute, Lodz, Poland

**Keywords:** PCSK9 inhibitors, cost-effectiveness, cardiovascular disease, dyslipidaemia treatment, statins

## Abstract

Dyslipidaemia is a complex disorder characterised by abnormal lipid levels in the blood, including cholesterol and triglycerides, and plays an important role in the development of atherosclerotic cardiovascular disease. Most risk factors for cardiovascular disease are modifiable, and dyslipidaemia is a key factor among them. It can result from a combination of genetic and environmental factors. A distinction is made between primary dyslipidaemia, which is mainly caused by inherited genetic changes, and secondary dyslipidaemia, which is due to underlying diseases or certain medications. The treatment of dyslipidaemia has evolved over the years. In the past, statins were the first choice, but newer drugs, such as proprotein convertase subtilisin-kexin type 9 (PCSK9) inhibitors, have gained prominence due to their effectiveness in lowering lipids. Although recent guidelines recommend PCSK9 inhibitors for high-risk patients and patients who cannot tolerate statins, their widespread use is limited because of cost. Several meta-analyses have confirmed the efficacy and safety of PCSK9 inhibitors and have shown a significant reduction in low-density lipoprotein (LDL) cholesterol levels. However, the long-term side effects and interactions with other risk factors for cardiovascular disease remain uncertain. In addition, cost-effectiveness analyses have shown mixed results, with some countries considering PCSK9 inhibitors to be cost-effective for certain patient groups, while others consider them less economical. Meanwhile, initial data from patients using PCSK9 inhibitors support the results of the clinical trials. To summarise, PCSK9 inhibitors represent a revolutionary solution for lowering LDL cholesterol, but their cost-effectiveness remains controversial. Despite the controversy, they offer clear benefits for high-risk patients and should therefore be considered in the treatment of dyslipidaemia.

## Introduction

Dyslipidaemia is a heterogeneous disorder characterised by abnormal lipid levels in the blood. These disorders are characterised by changes in the quantity, quality, or both of various lipid components, including cholesterol and triglycerides (1). They play an important role in the development of atherosclerotic cardiovascular diseases (CVD), such as coronary heart disease, acute myocardial infarction (MI), ischaemic stroke, peripheral arterial disease, and heart failure. The vast majority of risk factors for CVD are modifiable risk factors, such as diabetes mellitus, smoking, hypertension, obesity, and physical inactivity, with dyslipidaemia being one of the most important (2). Dyslipidaemia can result from a combination of genetic and environmental factors. A distinction is usually made between primary and secondary dyslipidaemia. Primary dyslipidaemia, of which familial hypercholesterolaemia is the most important and common, refers to lipid disorders that are primarily caused by inherited genetic changes. Traditionally, they are categorised into types I–V according to Frederickson's classification, based on the particles that show elevated levels in the blood. However, Frederickson's classification was first published in the 1960s. Today, patients in the 21st century need modern and updated classifications based on all aspects of cardiovascular risk and not just elevated lipid particles in the blood (3). Secondary dyslipidaemia or acquired dyslipidaemia occurs due to underlying diseases, lifestyle factors, or the use of certain medications. The most important risk factors for the development of secondary dyslipidaemia are obesity, diabetes mellitus, chronic kidney disease, chronic liver disease, and certain medications such as corticosteroids and diuretics taken over a long period of time (4). Even the new checkpoint inhibitors for oncological treatment have been observed to increase cholesterol levels, as indicated in the summary of product characteristics. Dyslipidaemia is therefore an increasingly attractive topic for the medical community, especially with the development of new therapeutic approaches (5). Until relatively recently, treatment with statins was the first choice for the treatment of dyslipidaemia. Nowadays, new drugs are being developed. The first new drug to come on the market was ezetimibe, which binds to the NPC1L1 transporter protein and thus inhibits fat absorption in the intestine (5). Proprotein convertase subtilisin-kexin type 9 (PCSK9) inhibitors were also developed: alirocumab and evolocumab as monoclonal antibodies, followed by the small RNA-interfering molecule inclisiran (6). All of these agents inhibit PCSK9, prolong low-density lipoprotein (LDL) receptor activation and lower LDL cholesterol levels (6).

In view of these therapeutic approaches, the European Atherosclerosis Society has published guidelines for the treatment of dyslipidaemia. The latest edition is from 2019 and the major innovation in these guidelines is the stratification of CVD risk for patients with primary dyslipidaemia using the revised SCORE system, called SCORE2 and SCORE-OP (7). In addition, these guidelines have revised the nomenclature of statin intolerance and place a strong emphasis on increasing statin treatment in patients previously discontinued due to non-specific, possibly statin-related symptoms. However, an important addition to the guidelines was also the inclusion of treatment recommendations based on new studies of PCSK9 inhibition that demonstrate the efficacy and safety profile of these drugs (8).

Although recent guidelines recommend their use in patients with a very high cardiovascular risk and in patients who cannot tolerate statins (which is not uncommon), PCSK9 inhibitors are not as widely used as statin treatment because of their cost (9). The aim of this review was to present the results of several meta-analyses conducted in the field of dyslipidaemia over the last 5 years, with a focus on PCSK9 inhibition. Another important aim of this paper was to discuss the results of the various studies in which PCSK9 inhibitors have been analysed in the context of cost-effectiveness with other lipid-lowering (LL) drugs. Instead of the 5-year period used for the aforementioned meta-analyses on safety and efficacy, we have chosen a shorter 3-year period (2020–2023) to get the best possible overview of the current market situation.

## Methods

For this literature review, PubMed, Scopus, Embase, and Web of Science were searched between October 2022 and 5 May 2023 using the keywords “PCSK9 inhibitors”, “efficacy”, “safety” and “cost-effectiveness”. We considered articles published in the 5-year period before the search. After a thorough assessment by two independent reviewers, we included 30 publications focusing on the efficacy and cost-effectiveness of PCSK9 inhibitors in the treatment of dyslipidaemia.

## Results

### Safety and efficacy of PCSK9 inhibitor treatment

PCSK9 inhibitors significantly reduced cardiovascular events, heart attacks, and ischaemic strokes compared to statins (Table 1). However, these consistent benefits were not observed when compared with ezetimibe (10, 11). Alirocumab and evolocumab showed a reduction in cardiovascular disease, myocardial infarction, and stroke compared to placebo, with varying effects on mortality (15, 20, 21). PCSK9 inhibitors showed a greater reduction in LDL-C levels compared to ezetimibe, with no significant differences between doses (14–16). The risk of myocardial infarction, stroke, heart failure, diabetes mellitus, neurocognitive events, and death was comparable to placebo in both lipid-lowering trials and clinical outcome trials (17, 18). The combination of dual lipid-lowering therapy with PCSK9 inhibitors and ezetimibe led to a significant reduction in total atheroma volume (12). PCSK9 inhibitors did not increase the risk of diabetes or cataracts (13, 25, 30, 31). In the FOURIER study, evolocumab showed a 31% relative risk reduction in venous thromboembolism compared to placebo. The expected relative risks for cholesterol reduction with PCSK9 inhibitors were reported as 0.851, 0.810, and 0.770 for a 20%, 30%, and 40% reduction, respectively (28). Lowering LDL-C levels was associated with a lower risk of major cardiovascular events, myocardial infarction, stroke, and death overall across different interventions and subgroups (27).

**Table 1:** Safety and efficacy of PCSK9 inhibitor treatment.

Year	Studies	Subjects	Treatment	Endpoint	Results
2023 (10)	49/1,709	66,068	Placebo or ezetimibe	Updated safety evaluation	No difference for all AEs (RR 1.023; 95% CI: 0.992–1.055) or serious AEs (RR 0.973; 95% CI: 0.944–1.003). Alirocumab was superior to control in serious AEs (RR 0.937; 95% CI: 0.896–0.980) but not evolocumab (RR 1.003; 95% CI: 0.963–1.054). Alirocumab had less diabetes-related AEs than control (RR 0.9137; 95% CI: 0.845–0.987), but this was not true for both treatments combined (RR 0.967; 95% CI: 0.914–1.023). No difference for neurocognitive and neurological AEs (RR 1.031; 95% CI: 0.913–1.163).
2022 (11)	49	1,724	Placebo with or without ezetimibe or placebo plus statin	Incidence of muscular adverse events	For myalgia, OR between treatment and control group was 0.91 but was not statistically significant. For myocardial infarction, OR was 0.68 but also not statistically significant.
2022 (12)	9/406	1,836	Ezetimibe, evolocumab, or alirocumab in addition to a statin vs. statins alone	Effects of ezetimibe, evolocumab, and alirocumab on coronary atherosclerosis using intravascular ultrasound	Significant favourable effect of evolocumab and alirocumab on total atheroma volume [standardised mean difference (SMD) −3.63; 95% CI: −4.44 to −2.83]. The addition of a PCSK9 inhibitor to a statin resulted in a significant reduction in the absolute change between baseline and follow-up for LDL-C (SMD −30.87; 95% CI: −39.29 to −22.45), total cholesterol (SMD −26.04; 95% CI: −36.49 to −15.58), and triglycerides (SMD −3.19; 95% CI: −5.56 to −0.82), but not HDL-C (SMD −1.14; 95% CI: −10.76 to 8.49). The addition of a PCSK9 inhibitor led to regression of plaque (SMD −1.01; 95% CI: −1.40 to −0.63) in patients with prior stain use, but not in statin-naive patients (SMD −0.94; 95% CI: −2.10 to 0.23). Evolocumab had no significant additional effect on the changes in fibrofatty plaque, fibrous plaque, necrotic core, or dense calcification.
2022 (13)	32/14,374	65,861	Placebo, standard care, or any other active lipid-lowering drugs	Safety	No evidence for new-onset diabetes mellitus (RR 0.99; 95% CI: 0.93–1.07), influenza-like symptoms leading to discontinuation (RR 1.5; 95% CI: 0.06–36.58), myalgia or muscular pain leading to discontinuation (RR 1.02; 95% CI: 0.22–4.74), any adverse events leading to discontinuation (RR 1.08; 95% CI: 0.99–1.19), neurocognitive events (RR 1.00; 95% CI: 0.87–1.15), cataract (RR 0.93; 95% CI: 0.8–1.07), or gastrointestinal haemorrhage (RR 0.60; 95% CI: 0.26–1.41). PCSK9 inhibitors increase a small absolute risk of ISR leading to discontinuation (15 more per 1,000; 95% CI: 11 to 20 more) in a 5-year time frame.
2021 (14)	7/993	926	Placebo	Efficacy and safety of alirocumab and evolocumab on familial hypercholesterolemia	PCSK9 inhibitors markedly decreased the LDL-C level by −49.14% (95% CI: −55.81 to −42.47%), and increased the level of HDL-C by 6.41% (95% CI: 4.09–8.73%), and Apo-A1 by 8.27 (95% CI: 3.38–13.16%). They also decreased the level of Apo-B by −38.09% (95% CI: −45.03–31.16%), non-HDL-C by −46.26% (95% CI: −53.45–39.06%), total cholesterol by −36.47% (95% CI: −42.09–28.84%), triglycerides by −10.26% (95% CI: −18.68 to −1.84%), and Lp(a) by −17.65% (95% CI: −24.75 to −10.55%). Comparable incidence of common AEs (RR 1.00; 95% CI: 0.82–1.22), serious AEs (RR 1.18; 95% CI: 0.39–3.54, and leading to treatment discontinuation (RR 1.23; 95% CI: 0.40–3.84) to placebo.
2021 (15)	45/1,820	97,297	Alirocumab, evolocumab, or bococizumab vs. placebo or ezetimibe	Efficacy and safety of PCSK9 inhibition in cardiovascular disease	Changes to lipidogram are reported, similar to ones seen in Merćep et al. (5). Unstable angina was less common in the alirocumab group (OR 0.69; 95% CI: 0.48–0.98), as was the frequency of myocardial infarction (OR 0.85; 95% CI: 0.76–0.95). There was no significant difference in the risk of unstable angina between evolocumab and control group (OR 0.66; 95% CI: 0.42–1.0). However, evolocumab was associated with lower risk of MI (OR 0.73; 95% CI: 0.65–0.82).
2020 (16)	10/10 (ODYSSEY)	1,709	Alirocumab vs. placebo or ezetimibe	Prevalence of discordant LDL-C/Lp(a) response to alirocumab at 24 weeks	The total prevalence of concordant response of LDL-C and Lp(a) to alirocumab was 78.5%. The prevalence of the two patterns of discordance was similar. There 12.6% patients who achieved LDL-C >35% reduction and Lp(a) ≤10% reduction. The opposite pattern of discordance [LDL-C ≤35% reduction and Lp(a) >10% reduction] was slightly less common (8.9%). The total prevalence of discordant responses was 21.5%.
2021 (17)	36/65 (statins) 5/53 (PCSK9 inhibitors)	204,918 (statins) 76,140 (PCSK9 inhibitors)	Statins	Risk of haemorrhagic stroke	For all patients and any dose, there was a significant increased risk of haemorrhagic stroke with statins compared with control and high-dose statins compared with low-dose statins (0.42% vs. 0.36%; RR 1.15; 95% CI: 1.00–1.32). There was not a significant increased risk of haemorrhagic stroke with PCSK9 inhibitors added to maximally tolerated statins compared with maximally tolerated statins alone (0.09% vs. 0.09%; RR 0.93; 95% CI: 0.58–1.51). For statins, but not for PCSK9 inhibitor, risk is magnified in a medication dose-dependent and type of vascular brain injury-dependent manner.
2021 (18)	7/211	57,440	Placebo or standard therapy	Effects of PCSK9 inhibitors on brain stroke prevention	PCSK9 inhibitors significantly reduced the total brain stroke risk in comparison with controls (RR 0.77; 95% CI: 0.67–0.88) and ischaemic brain stroke (RR 0.76; 95% CI: 0.66–0.89), but not haemorrhagic brain stroke (RR 1.00; 95% CI: 0.66–1.51). Also, PCSK9 inhibitors did neither reduce cardiovascular mortality (RR 0.95; 95% CI: 0.84–1.07) nor increase the incidence of neurocognitive impairment (RR 1.02; 95% CI: 0.81–1.29).
2020 (19)	41/1,360	64,107	Placebo and/or ezetimibe and/or other lipid-lowering therapy	Effects of PCSK9 inhibitors on the serum Lp(a) levels	PCSK9 inhibitors reduced Lp(a) levels by −26.7% (95% CI: −29.5% to −23.9%). There was no difference in the rate of treatment-related adverse events between the intervention and comparator arms (RR 1.01; 95% CI: 0.98–1.03).
2020 (20)	10/57	50,568	Statins or ezetimibe		Compared to statins, the treatment with PCSK9 inhibitors was associated with a statistically significant decrease in cardiovascular events (RR 0.87; 95% CI: 0.83–0.91), non-fatal myocardial infarction (RR 0.86; 95% CI: 0.78–0.96), and ischaemic stroke (RR 0.75; 95% CI: 0.64–0.87). The same was not always true in comparison with ezetimibe. PCSK9 inhibitors were not associated with decrease of cardiovascular death (RR 0.96; 95% CI: 0.85–1.08) or all-cause mortality (RR 0.95; 95% CI: 0.86–1.05).
2020 (21)	24/1,218	60,997	Background lipid-lowering treatment or lifestyle counselling	Efficacy and safety	Alirocumab compared with placebo decreased the risk of CVD events (OR 0.87; 95% CI: 0.80–0.94), mortality (OR 0.83; 95% CI: 0.72–0.96), myocardial infarction (OR 0.86; 95% CI: 0.79–0.94), and for any stroke (OR 0.73; 95% CI: 0.58–0.91). Results for evolocumab compared to placebo were as follows: for CVD: OR 0.84 (95% CI: 0.78–0.91); for mortality: OR 1.04 (95% CI: 0.91–1.19); for MI: OR 0.72 (95% CI: 0.64–0.82); for any stroke: OR 0.79 (95% CI: 0.65–0.94). Compared to active treatment, both alirocumab and evolocumab effects were smaller or non-significant.
2021 (22)	8	1,602	Ezetimibe	Efficacy of ezetimibe vs. PCSK9 inhibitors in patients not on statins	PCSK9 inhibitors lowered LDL-C levels significantly more than ezetimibe (MD −36.5; 95% CI: −38.3 to −34.7). In the statin intolerant subgroup, PCSK9 inhibitors showed significantly greater reduction in LDL-C levels compared with ezetimibe (MD −36.1; 95% CI: −39.2 to −33.1). There were no significant differences in LDL-C reduction between different PCSK9 inhibitor dosages (140 mg once every 2 weeks vs. 420 mg once every 4 weeks) (MD −1.87; 95% CI: −4.45 to 0.71).
2020 (23)	38/53	90,794	Placebo	The effect of PCSK9 inhibitors on the risk of serious AEs or death using ClinicalTrials.gov database	For PCSK9 inhibitors, the risk of myocardial infarction was statistically comparable to placebo in the LL (OR 0.92; 95% CI: 0.64–1.30) and CO trials (OR 0.88; 95% CI: 0.64–1.22). Same was true for stroke/TIA in the LL (OR 1.32; 95% CI: 0.83–2.09) and clinical outcome trials (OR 0.97; 95% CI: 0.79–1.19), for heart failure in the LL (OR 0.96; 95% CI: 0.60–1.56) and clinical outcome trials (OR 0.99; 95% CI: 0.84–1.17), diabetes mellitus in the LL (OR 1.17; 95% CI: 0.75–1.82) and CO trials (OR: 1.05; 95% CI: 0.98–1.13), risk for neurocognitive events in the LL (OR 1.19; 95% CI: 0.76–1.86) and CO trials (OR 1.22; 95% CI: 0.70–2.11), and the risk of death in the LL (OR 0.80; 95% CI: 0.51–1.24) and CO trials (OR 0.99; 95% CI: 0.84–1.17). However, for evolocumab, the risk of mortality was 1.18 (95% CI: 0.46–3.02) in LL trials and 1.12 (95% CI: 1.00–1.25) in the clinical outcome trial FOURIER.
2020 (24)	8/524	1,759	Statin or statin and ezetimibe	Effect of dual lipid-lowering therapy (statin + non-statin drugs) on coronary atherosclerosis regression	Dual lipid-lowering therapy was associated with a significant reduction in total atheroma volume both ezetimibe group (−4.0 mm^3^; 95% CI: −6.5 to −1.5) and PCSK9 inhibitor group (−3.9 mm^3^; 95% CI : −6.0 to −1.7). A 10% decrease in LDL-C or non-HDL-C levels was associated, respectively, with 1.0 and 1.1 mm^3^ regressions in total atheroma volume.
2020 (25)	8/279	72,298	Ezetimibe	Risk of new-onset diabetes	PCSK9 inhibitors do not increase the risk of diabetes (RR 0.99; 95% CI: 0.92–1.07) and same was true for ezetimibe (RR 1.05; 95% CI: 0.95–1.15).
2020 (26)	30	59,026	Alirocumab vs. evolocumab	Indirect comparison of the efficacy and safety of alirocumab vs. evolocumab	Alirocumab was associated with reduction in all-cause death (RR 0.80; 95% CI: 0.66–0.97) but not in cardiovascular death (RR 0.83; 95% CI: 0.65–1.05) and no significant differences in myocardial infarction (RR 1.15; 95% CI: 0.99–1.34), stroke (RR 0.96; 95% CI: 0.71–1.28) or coronary revascularization (RR 1.13; 95% CI: 0.99–1.29). Alirocumab was associated with a 27% increased risk of injection site reaction compared to evolocumab; however, no significant differences were found in terms of treatment discontinuations, systemic allergic reaction, neurocognitive events, ophthalmologic events, or new-onset of or worsening of pre-existing diabetes
2020 (27)	2 (FOURIER and ODYSSEY)	46,488	Placebo	Effects of PCSK9 inhibitors on venous thromboembolism	In the FOURIER trial, the hazard ratio (HR) for venous thromboembolism with evolocumab was 0.71 (95% CI: 0.50–1.00), while in the ODYSSEY trial, the HR was 0.67 (95% CI: 0.44–1.01). A meta-analysis of the two trials demonstrated a statistically significant 31% relative risk reduction in VTE with PCSK9 inhibition compared with placebo (HR 0.69; 95% CI: 0.53–0.90).
2019 (28)	4 (FOURIER, SPIRE 1 and 2, ODYSSEY)	—	Evolocumab, bococizumab and alirocumab	Effects of PCSK9 inhibitors on the risk of stroke, an updated meta-regression approach	According to the results of the effects of PCSK9 inhibitor treatments on cholesterol levels, authors suggest that the expected an RR of 0.851, 0.810, and 0.770 will be seen for a 20%, 30%, and 40% reduction in total cholesterol, respectively.
2019 (29)	12/1,416	131,978	Statin or ezetimibe	Cardiovascular benefits of high-dose statin, ezetimibe + statin, and PCSK9 inhibitors + statin treatments in secondary prevention patients	Major cardiovascular event risk was associated with an RR of 0.86 (95% CI: 0.81–0.90), 0.90 (95% CI: 0.83–0.96), and 0.94 (95% CI, 0.92–0.96); myocardial infarction risk was associated with an RR of 0.85 (95% CI: 0.74–0.95), 0.88 (95% CI: 0.80–0.96), and 0.90 (95% CI: 0.85–0.95); stroke risk was associated with an RR of 0.86 (95% CI: 0.76–0.95), 0.83 (95% CI: 0.66–0.99), and 0.93 (95% CI: 0.85–1.02); all-cause death risk was associated with an RR of 0.90 (95% CI: 0.79–1.00), 0.86 (95% CI: 0.55–1.17), and 0.98 (95% CI: 0.92–1.04); and cardiovascular death risk was associated with an RR of 0.88 (95% CI: 0.75–1.00), 0.99 (95% CI: 0.85–1.14), and 0.99 (95% CI: 0.93–1.04) per 1 mmol/L reduction of the LDL-C level, respectively, in the subgroups with high-dose statins, ezetimibe-statin, and PCSK9 inhibitor-statin as intervention.
2019 (30)	5/398	83,492	Placebo	Risk of cataract development	PCSK9 inhibitor therapy was not associated with an increased risk of cataracts (OR: 0.96; 95% CI: 0.85–1.08)
2018 (31)	20/133	68,123	Placebo	Manifestation of diabetes	Patients treated with PCSK9 inhibitors had an absolute increase of incidents diabetes (weighted mean difference) of 1.88 mg/dl (95% CI: 0.91–2.68), which was significantly different from placebo (standardised mean difference 0.166%; 95% CI: 0.143–0.188). Regarding HbA1c levels, compared with baseline, patients treated with PCSK9 inhibitors had a weighted mean difference of 0.032% (0.011–0.050; standardised mean difference 0.096%; 95% CI: 0.074–0.119).

AE, adverse event; RR, risk ratio; OR, odds ratio; MD, mean deviation; TIA, transient ischemic attack; ISR, in-stent restenosis; VTE, venous thromboembolism.

The analysis showed no significant difference in overall or serious adverse events compared to the control group. Alirocumab showed superiority in reducing serious adverse events and had fewer diabetes-related events than the control group (21, 26). No significant differences were observed for evolocumab (22). There was also no significant difference in neurocognitive and neurological adverse events (23). The odds ratios for myalgia and myocardial infarction did not reach statistical significance. Both evolocumab and alirocumab had a favourable effect on total atheroma volume (24). PCSK9 inhibitors significantly reduced LDL-C levels and had a favourable effect on lipid profiles (19, 29). There was no evidence of new-onset diabetes, flu-like symptoms, or other adverse events leading to discontinuation of treatment (13). PCSK9 inhibitors did not increase the risk of injection site reactions over a 5-year period (13).

### Cost-effectiveness of the PCSK9 inhibitor treatment

The comprehensive analysis of cost-effectiveness in different regions, including Germany, the United Kingdom, Canada, Spain, China, Saudi Arabia, and the Russian Federation, underlines the favourable economic profile of PCSK9 inhibitors, particularly evolocumab and alirocumab, in the context of cardiovascular risk management (Table 2) (32–39). Comparison with other lipid-lowering agents, including icosapent ethyl, fibrates, fenofibrate, and ezetimibe, consistently shows a more favourable cost–benefit ratio for PCSK9 inhibitors (32–39). These results suggest that the inclusion of evolocumab and alirocumab in treatment strategies not only holds promise for optimising clinical outcomes (CO) in high-risk cardiovascular patients, but is also consistent with cost-effective considerations, making them potentially valuable additions to therapeutic protocols.

**Table 2:** Cost-effectiveness of the PCSK9 inhibitor treatment.

Year	Methodology	Country	Treatment	Cost	QALYs
2022 (32)	Data derived from cardiovascular outcome trials for statin combinations with other drugs.	Germany	Icosapent ethyl^a^	14,732€	0.81
			Fibrates^a^	−10,516	0.63
			Ezetimibe^b^	−5,796	0.61
			Icosapent ethyl^b^	14,333	0.99
			Evolocumab^b^	62,722	0.55
			Alirocumab^b^	87,002	0.87
2022 (33)	Simulation with Markov cohort model for statin combinations with other drugs.	UK	Icosapent ethyl^a^	15,421 Ł	0.79
			Fenofibrate^a^	−6,127	0.62
			Icosapent ethyl^b^	12,981	0.98
			Ezetimibe^b^	−2,529	0.60
			Evolocumab^b^	45,279	0.53
			Alirocumab^b^	46,375	0.86
2022 (34)	Markov cohort state transition model in ASCVD patients with prior MI and baseline LDL-C ≥1.8 mmol/L for adding evolocumab to statin therapy.	Canada	Evolocumab^b^	66,453 CAD	1.00
2021 (35)	A 20-year Markov model comparing evolocumab with standard of care vs standard of care alone.	Spain	Evolocumab^b^	289,677€	1.00
2022 (36)	Markov model based on data from clinical trials, claims databases, and published literature to compare evolocumab with statins vs. placebo combined with statins. Adult patients with ASCVD and LDL-C levels >70 mg/dl after the maximum tolerable dose of statin therapy.	China	Evolocumab^b^	18,714 CNY	1.25
2022 (37)	Markov model of evolocumab compared with ezetimibe, both added to background statin therapy in patients with recent ACS events and LDL-C levels ≥100 mg/dl	China	Evolocumab^b^	115,782 CNY	1.33
2022 (38)	Markov model in patients with clinically evident atherosclerotic cardiovascular disease and baseline LDL-C ≥70 or ≥100 mg/dl, adding evolocumab to a maximally tolerated statin, with or without ezetimibe.	Saudi Arabia	Evolocumab^b^ + s	60,708 $	1.00
			Evolocumab^b^ + s + ez	41,757	1.00
2023 (39)	A Markov model to characterise the development of atherosclerotic heart disease in patients with very high CV risk.	Russian Federation	PCSK9 inhibitors and inclisiran^b^	3.6M RR	1.00

^a^ Primary.

^b^ Secondary.

ASCVD, atherosclerotic cardiovascular disease.

## Discussion

Drugs for the treatment of dyslipidaemia are among the most commonly used medicines. According to the National Ambulatory Medical Care Survey published by the Centres for Disease Control and Prevention in 2019, medications to treat dyslipidaemia were the second most frequently mentioned medications in primary care practices in the United States, surpassed only by analgesics, while atorvastatin was the second most frequently used prescription medication (40). For decades, statins were synonymous with the treatment of dyslipidaemia, but in recent years the new generation of lipid-lowering drugs has become more widely used as a result of new research findings and general awareness of the potential adverse effects of statins (although these are usually vastly overestimated). In second place in the treatment of dyslipidaemia are currently the PCSK9 inhibitors—alirocumab, evolocumab, and inclisiran, which is not strictly a PCSK9 inhibitor, but is often included in the same group of drugs. Numerous research papers have been published on the safety and efficacy of PCSK9 monoclonal antibodies, and all these papers have in common that they have demonstrated the efficacy in lipid lowering and the good safety profile of these drugs (41). Another important point that is often emphasised as a major advantage is their compliance, as they are administered once or twice a month. However, to date, no study has been published that demonstrates better compliance compared to standard daily oral statin treatment or combined treatment with statin + ezetimibe or bempedoic acid (42). In addition, recent results have confirmed the efficacy of PCSK9 inhibitors in reducing major cardiovascular events and have also shown the benefit of treatment with PCSK9 inhibitors in schematic stroke (43). Regarding the safety profile of PCSK9 inhibitors, most studies showed a very good safety profile; however, the targets were evaluated in the relatively short term so that potential long-term adverse effects could not yet be assessed. This brings us to the main problem in interpreting the previously published results on PCSK9 inhibition. In our study, we found that the number of published clinical trials is relatively low—106 compared to 62 meta-analyses—with new meta-analyses being published monthly, while there are currently only 76 active and recruiting clinical trials, based on the web source clinicaltrials.gov. Most trials focus on PCSK9 inhibition and serious cardiovascular events, but we can see a trend in pharmacology continuing in the lipid-lowering field—entirely new indications for PCSK9 inhibitors are being tested (44). On the other hand, published work to date has not yet shown promising results in patients with other risk factors for serious cardiovascular events, such as diabetes mellitus, chronic kidney disease, or other non-communicable diseases. The question might be: Have we jumped on the PCSK9 bandwagon too early? Why is there a sudden need for new potential indications for medications whose long-term side effects and interactions with the pathophysiology of other comorbidities are still unknown. Our patients not only have dyslipidaemia, but very often other risk factors that lead to serious cardiovascular events as well. One positive effect of the 21st century is the willingness of the medical community to publish real-world data on certain topics, and PCSK9 inhibition is no exception. There is an increasing number of publications based solely on real-world data confirming the results of clinical trials and meta-analyses of PCSK9 inhibitors (45–48).

An important issue that has not yet been mentioned is the cost-effectiveness of PCSK9 inhibitors. An important factor in treatment with PCSK9 inhibitors is their relatively high cost. The initial cost–benefit analyses all agreed that PCSK9 inhibitor therapy is not cost-effective, with reports ranging from developed countries to developing countries (49–51). Nevertheless, the price of evolocumab and alirocumab has fallen in recent years (52). However, even with this price reduction, some cost-effectiveness analyses have not shown positive results for the general use of PCSK9 inhibition. One of the most recent published analyses from the UK showed quality-adjusted life years (QALY) of 0.53 and 0.86 for evolocumab and alirocumab, respectively, at a cost of approximately £45,000. With a National Health Service willingness to pay £30,000/QALY, PCSK9 inhibitors do not appear to be cost-effective (33, 53). On the other hand, a Swedish analysis showed that PCSK9 inhibitors are cost-effective in the secondary prevention of myocardial infarction in combination therapy for very high-risk patients (54).

## Conclusion

When we talk about PCSK9 inhibitors, we must all agree that they represent a revolutionary solution for lowering LDL cholesterol that the medical community awaited for decades after the introduction of statins. Finally, we now have clinical trial results and real-world data confirming the efficacy of the treatment regimen and the safety profile of evolocumab and alirocumab. For other novel treatment options based on PCSK9 inhibition, studies demonstrating the potential cardiovascular benefit or real-world data are still lacking. It must be mentioned that these new drugs are also expensive. When we talk about the costs and expenses of evolocumab and alirocumab, we can safely say that the cost-effectiveness of PSCK9 inhibitors is still very controversial, and the interpretation of the analysis results depends on the thresholds of the statutory health insurance funds. However, their benefit in high-risk patients is absolutely clear, regardless of the costs.
